# Supplementary material for: Pathophysiological Significance of Neutrophilic Transfer RNA-Derived Small RNAs in Asymptomatic Moyamoya Disease
Source: Cells. 2021 May 1;10(5):1086. doi: 10.3390/cells10051086 (PMC8147334; doi:10.3390/cells10051086)

2D Structure	Local AU	Position

```
69                                8mer-1a       96
 5'-caGAAACCCTATAAATGTA-AATGTGGGa-3' utr
      |||  ||   |:    |:   |   |||||||
 3'-ucCUUAGGAUUGGCGAUCUGGUACACCCu-5' tRF
                16151413    7 6 5 4 3 2
              3' Pairing       Seed
```

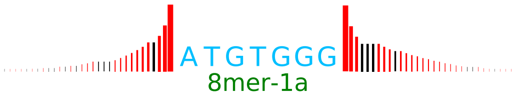

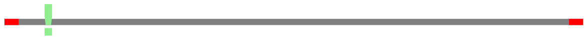

2D Structure	Local AU	Position

```
149                                8mer-1a       179
 5'-agaGATGCTGTATGATATAAGGAATGTGGGa-3' utr
       :||  ||:   :|  ||  |      |||||||
 3'-uccUUAGGAUUGGCGAUCU--GGUACACCCu-5' tRF
                16151413      7 6 5 4 3 2
              3' Pairing          Seed
```

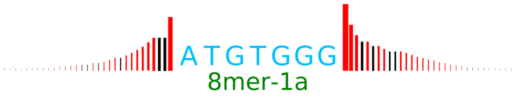

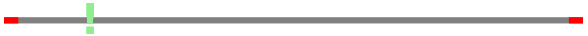

Supplement: Supplementary file 1 [file cells-10-01086-s001.zip › cells-1149161 3rd supplementary materials/Data Supplements/Data Supplement V.zip!/Data supplement 6-mRNAs targets of tsRNAs/tsRNA-Glu-TTC-010/Diagrams/tRF-Glu-TTC-010_vs_ENST00000293725(ZNF563).pdf]
